# Advancing Immunoassay Precision: A Novel Preanalytical Method for Enhancing Thyroglobulin Measurement in the Presence of Tg Antibodies

**DOI:** 10.3390/ijms252413252

**Published:** 2024-12-10

**Authors:** Ilona Đorić, Aleksandra Todorović, Marija Gnjatović, Snežana Golubović, Miloš Žarković, Jelena Janković Miljuš, Tijana Išić Denčić, Sonja Šelemetjev

**Affiliations:** 1 Institute for the Application of Nuclear Energy INEP, University of Belgrade, Banatska 31b, 11080 Belgrade, Serbia; marecko@inep.co.rs (I.Đ.); aleksandra.todorovic@inep.co.rs (A.T.); marijad@inep.co.rs (M.G.); sneza@inep.co.rs (S.G.); jelenaj@inep.co.rs (J.J.M.); tijana@inep.co.rs (T.I.D.); 2 Clinic for Endocrinology, Diabetes and Metabolic Diseases, Clinical Centre of Serbia, Doktora Subotića 13, 11000 Belgrade, Serbia; milos.zarkovic@med.bg.ac.rs

**Keywords:** Tg, Tg immunoassays, IRMA, preanalytical treatment, TgAb interference, DTC follow-up, recurrent DTC

## Abstract

Thyroglobulin (Tg) is a reliable marker for detecting recurrence in differentiated thyroid cancer (DTC) patients, but frequently occurring Tg antibodies (TgAbs) can hinder accurate measurement. We aimed to develop a preanalytical protocol for precise Tg detection in TgAb presence using the immunoradiometric assay (IRMA) platform. This study involved forty-five patients who underwent IRMA Tg and radioimmunoassay (RIA) TgAb measurements, including two patients monitored for recurrence and one with confirmed recurrence. All three had undetectable Tg levels. We evaluated three preanalytical methods in aiming to separate Tg from TgAbs: buffer only (Protocol 1), micro-spin filters only (Protocol 2), and a combination of both (Protocol 3). All preanalytical protocols showed high concordance with the original test (r = 0.981, 0.985, 0.971, respectively, *p* < 0.001), regardless of TgAb values. Protocols 1 and 3 yielded higher Tg levels than the original test (*p* < 0.001), especially in the group with a high TgAb titer. Protocol 1 managed to detect Tg in two patients under follow up with initially unmeasurable Tg and high TgAb titers and in one confirmed recurrent case. Sample pre-processing positively influenced Tg detection in TgAb-positive cases. These preanalytical approaches show promise, but further testing with larger sample sizes and more investigated conditions is warranted.

## 1. Introduction

Differentiated thyroid cancer (DTC) accounts for nearly 95% of all thyroid malignancies overall [1]. Treatment typically involves total thyroidectomy and/or radioactive iodine ablation, yielding a favorable prognosis for most patients [2,3]. However, 10–20% will experience recurrence, with some metastases occurring even well after 5 and 10 years from initial treatment [4,5]. Therefore, lifelong follow up is recommended for all patients, especially the higher-risk categories [6,7]. Thyroglobulin (Tg), a glycoprotein produced exclusively by the follicular thyroid cells, serves as a reliable tumor marker for monitoring and managing patients with DTC after surgery [8]. As DTCs retain the expression of thyroid-specific markers, the presence of Tg in the serum of patients surgically treated for thyroid cancer indicates persistent or recurrent disease. Routine follow up of DTC includes Tg measurement as the initial test for detecting recurrence, measured every 6 to 12 months or more often in case of high-risk cases [6,7], making this assay an indispensable tool in long-term DTC follow up.

In most clinical laboratories, the levels of Tg are measured via immunoassays, which encompass competitive radioimmunoassays (RIAs) and immunometric assays (IMAs), further divided into immunoradiometric (IRMAs), chemiluminescent (CLIAs), and enzyme immunoassays (ELISAs). In detecting DTC recurrence, any level of Tg higher than 1 µg/mL is considered positive for recurrence [6,7]; therefore, functional sensitivity of the Tg assay is essential. Both RIA and IRMA methods offer superior sensitivity and allow for the detection of low concentrations of substances [9]; however, due to safety concerns with handling radioactive materials, enzyme-based detection IMAs (such as ELISAs) are more commonly used in routine practice. However, while being favored for their sensitivity, simplicity, and extensive validation [10], Tg IMAs are prone to interference from, predominantly, antithyroglobulin antibodies (TgAbs) and heterophile and human anti-animal antibodies, lowering their sensitivity [11,12]. The mechanism of interference is hypothesized to involve steric hindrance or epitope masking due to the formation of antigen–antibody complexes with Tg, and it results in false negative Tg measurements [13]. The RIA is less affected by Ab interference, but it is still not devoid of its influence, and both over- and underestimation of Tg RIA levels can occur in the setting of the presence of TgAbs [14]. Our experience with the IRMA test for measuring Tg, particularly at very low Tg concentrations, suggests that underestimation, rather than overestimation, of Tg values occurs frequently in the context of Tg autoantibodies.

The solutions for the issues arising with immunoassays for Tg in DTC follow up have included the use of the TgAb as a surrogate marker for Tg and the implementation of liquid chromatography–mass spectrometry (LC-MS/MS) analyses of selected TgAb-positive DTC patients. The use of surrogate TgAbs has not been universally recommended in guidelines of the major thyroid societies due to the inability to standardize TgAb levels, the variability of TgAb assays, and the lack of direct proportion with the levels of Tg [15,16,17,18]. LC-MS/MS is a robust measurement analysis, unaffected by interference, but its commercial use is limited due to the complexity of the method, the cost of analysis, and low sample throughput [19].

A more successful way of overcoming these obstacles has emerged through enhancing the immunoassays by utilizing various pretreatment methods to disrupt the bond between Tg and TgAbs. This involves exploring different buffer formulations with varying levels of chaotropic agents, detergents, and salt concentrations and making pH adjustments to effectively dissociate the Tg-TgAb complexes. In tackling the interference caused by TgAbs, the group from the R&D of FUJIREBIO INC. and R&D of Advanced Life Science Institute, Inc., Tokyo, Japan, created an immunoassay that utilizes a fully automated chemiluminescent enzyme system [20]. This approach includes an efficient specimen pretreatment process designed to inactivate TgAbs in blood and concluded that the novel assay outperforms second-generation sandwich immunoassays, providing accurate Tg concentrations, even in cases where the TgAb is present. This procedure is protected by a patent publication in the US since 2021 [21].

Our study aimed to develop a protocol for the preanalytical processing of serum to enable precise detection of Tg in patients with low Tg concentrations and the presence of TgAbs. To achieve this goal, we tested three protocols. The first protocol involved selecting the best buffer system to disrupt the Tg-TgAb complex, allowing the release of Tg for more accurate measurement. The second protocol employed spin filters to separate proteins based on molecular weight, allowing immunoglobulins, which have a smaller molecular mass, to pass through, while retaining Tg and allowing the free Tg to be measured. The third protocol combined the first two, disrupting the Tg-TgAb bond and subsequently removing antibodies from the serum using filter columns. This improvement aims to benefit patients undergoing follow up for the early detection of thyroid cancer recurrence and to assist endocrinologists in the accurate interpretation of diagnostic results. The pretreatment was coupled with the IRMA platform, ensuring high throughput, cost effectiveness, and reliability for standardization and clinical laboratory use, but it was developed to be compatible with all immunometric platforms on the market.

## 2. Results

### 2.1. Interlaboratory Comparison of Tg IVD Assays

**Figure 1:**
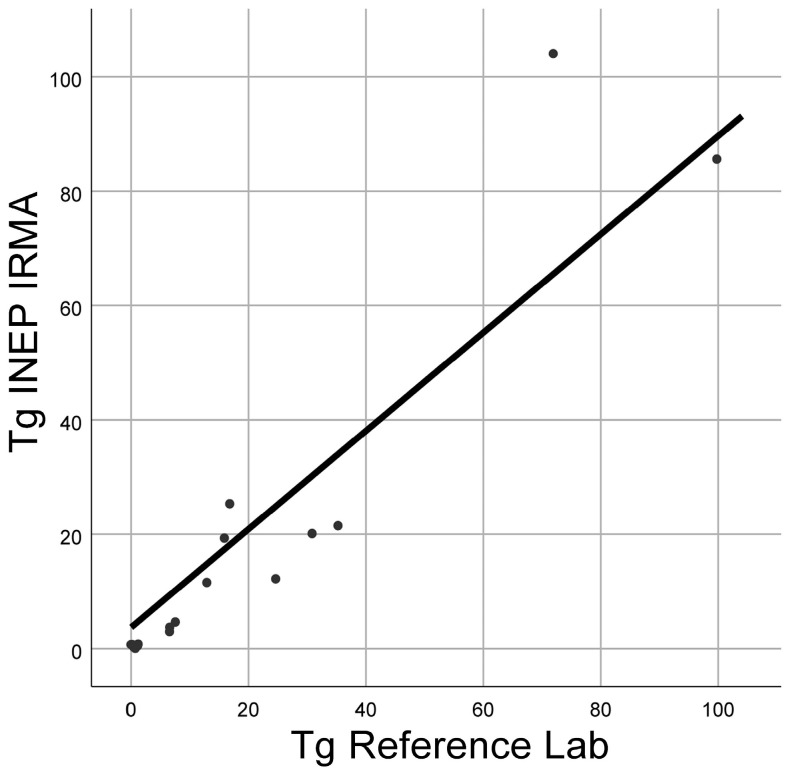
Concordance between Tg INEP and Tg reference lab measurements, both measured in µg/L.

The Tg IRMA INEP test’s performance was compared to Tg’s test performance measured in the biochemical laboratory of the Military Medical Academy (Tg reference lab). The comparison included 17 randomly selected samples. The results showed that there was no statistically significant difference between the Tg levels measured by the aforementioned tests (Wilcoxon signed-ranks test, *p* = 0.136), and the measured concentrations of Tg showed a high positive correlation (Spearman’s rho = 0.938, *p* < 0.000; Figure 1). Having confirmed that the Tg IRMA INEP test presented high concordance in interlaboratory comparison, we used this immunoassay in all further Tg measurements.

### 2.2. Development of Preanalytical Protocols for Tg IRMA Measurement

To develop a streamlined preanalytical treatment method for samples, we designed three different protocols as follows.

(a) Protocol 1: preanalytical processing of serum using a buffer system. This method is based on the principle that buffer systems disrupt antigen–antibody bonds. Selected buffer systems were chosen based on previous research, experimental experience with ELISA test optimization, and the findings of other groups addressing similar issues [22,23,24,25].

In this study, we tested five buffer systems that differed in pH values, salt concentration, and percentage of sugar component, and two different dilutions of each buffer. Three buffers were immediately excluded due to producing false positive readings in the IRMA test. The remaining two buffers (arbitrarily named 1A and 1B) were retained for further testing and evaluated at two different concentrations (10% and 50%), resulting in four testing sets: 1A with 10%, 1A with 50%, 1B with 10%, and 1B with 50%. We tested these buffers on 12 randomly selected serum samples to see which of the two solutions increased Tg levels more effectively. The solution with 10% 1B buffer was selected for downstream analysis as it increased Tg values in 72.2% of cases compared to the baseline test measurements, while the solution with 10% 1A buffer increased the Tg level only in 10% of tested cases.

We established the influence of the buffer used in Protocol 1 on the measurement of thyroglobulin using known concentrations of recombinant human thyroglobulin (rhTg). We compared the differences in Tg measurement with the INEP IRMA test between different dilutions of rhTg in a standard dilution buffer with the same rhTg dilutions with the addition of a dissociation buffer in 10% (Protocol 1). The results are shown in Figure S1. Statistical analysis of the results showed that there is no significant difference between the tested groups (Wilcoxon signed-rank test, *p* = 1.000), meaning that the addition of the buffer from Protocol 1 does not influence the measurement of Tg.

(b) Protocol 2: preanalytical processing of serum using micro-spin filters. Preanalytical processing of serum using micro-spin filter (size exclusion with a cut off of 200 kDa) operates by separating Tg from smaller molecules.

(c) Protocol 3: preanalytical processing of serum using a combination of buffer system and micro-spin filters. Preanalytical processing of serum was performed by using a combination of the buffer system and micro-spin filters, combining both approaches by first disrupting the antigen–antibody bonds and then separating the molecules.

### 2.3. Influence of Preanalytical Treatment Protocols on Tg IRMA Measurement

After developing the streamline for preanalytical treatments, we next evaluated the effect of three preanalytical protocols for sample processing on the concentration of Tg measured via the IRMA method. In total, 46 cases were processed with Protocol 1 (buffer only), 39 cases were processed with Protocol 2 (filter only), and 43 cases were processed with Protocol 3 (both buffer and filter).

**Figure 2:**
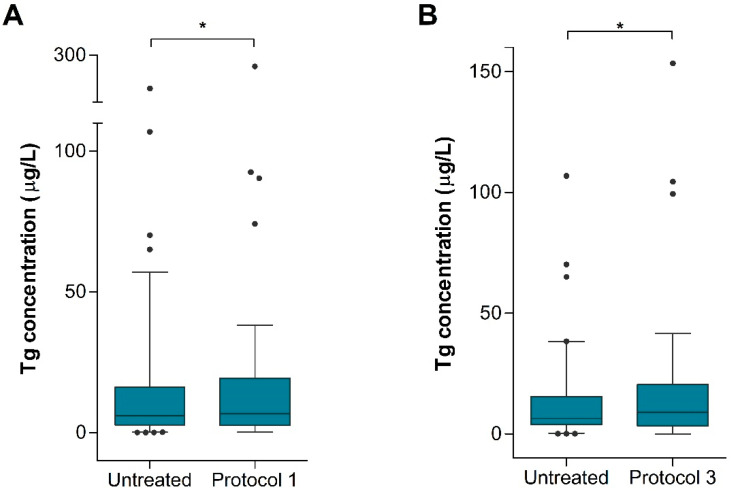
Influence of preanalytical processing on Tg concentration. Compared to the untreated samples, Tg levels were significantly changed after treatment by (**A**) Protocol 1 and (**B**) Protocol 3. Boxes represent the median with IQR. * *p* < 0.05.

After comparing Tg concentrations before and after each preanalytical protocol, we perceived a statistically significant difference in Tg levels in untreated vs. Protocol 1-treated serum (Wilcoxon signed-rank test, *p* = 0.009, *n* = 46). Additionally, we observed a difference in Tg levels between untreated serum and serum treated by Protocol 3 (Wilcoxon signed-rank test, *p* = 0.000, *n* = 43). Protocol 2 alone did not significantly affect Tg levels (Wilcoxon signed-rank test, *p* = 0.396, *n* = 39). Statistically significant results are represented in Figure 2.

When we analyzed the correlations between Tg levels measured before and after pre-processing, we observed significant positive correlations between all tested protocols (Table 1). The observed Spearman correlations between all three protocols indicate that as Tg measurements increase across all samples, this trend is consistently reflected in each of the protocols. In other words, the rise in Tg values is parallel across the three protocols, suggesting a consistent relationship in the way each protocol measures Tg.

### 2.4. Effects of Preanalytical Processing on Tg Measurement in Patients Positive for Tg Antibodies

After establishing that the tested experimental protocols affect Tg, we proceeded to the next phase of this study and analyzed the effect of pre-processing on Tg levels in the serum of patients positive for Tg antibodies. In order to present the effect more robustly, we divided the samples into three groups based on TgAb levels (negative, low, and high), as described in the Materials and Methods section.

**Figure 3:**
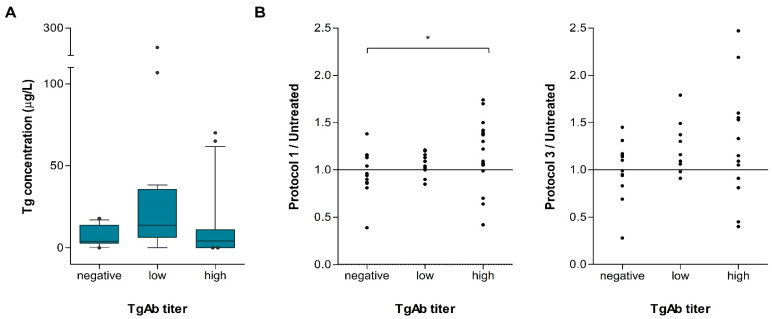
Tg concentration in untreated serum samples and effects of serum pre-processing grouped according to TgAb titers. (**A**) Tg concentration in untreated serum samples. Boxes represent the median with IQR. (**B**, **left**) Effect of Protocol 1 pre-processing; the values on the *y*-axis represent the ratio of Protocol 1-treated Tg levels to Tg in untreated serum. (**B**, **right**) Effect of Protocol 3 pre-processing; the values on the *y*-axis represent the ratio of Protocol 1-treated Tg levels to Tg in untreated serum. * *p* < 0.05.

We first observed that the levels of Tg were lower in the group without TgAbs compared to the low TgAb group, and they also had a smaller concentration range. When the two groups that were positive for TgAbs were compared, a higher amount of Tg was detected in the low TgAb group compared to the high TgAb group (Figure 3A).

To present the effects of the treatments, we normalized the concentration of Tg treated with different protocols to the values of Tg concentrations in untreated serum; thus, the ratio values of >1 represented the increase in Tg levels caused by the treatment. Figure 3B shows the ratios of Tg levels in samples treated by either Protocol 1 or Protocol 3 in groups with different TgAb levels. The results showed a trend of increasing Tg levels after treatment by Protocol 1 with the rising levels of TgAbs (Figure 3B, left). Statistical analyses showed that Protocol 1 pretreatment significantly alters Tg levels in samples that are positive for TgAbs (Kruskal–Wallis test, *p* = 0.050) and that the difference between the group negative for TgAbs and with high TgAb levels is predominantly driving this difference (post hoc pairwise comparison, *p* = 0.016). The same trend of Tg concentration change was also observed after treatment by Protocol 3 but did not exhibit statistical significance (Figure 3B, right).

### 2.5. Effects of Preanalytical Processing on Tg IRMA Measurement in Patients Treated for DTC

**Figure 4:**
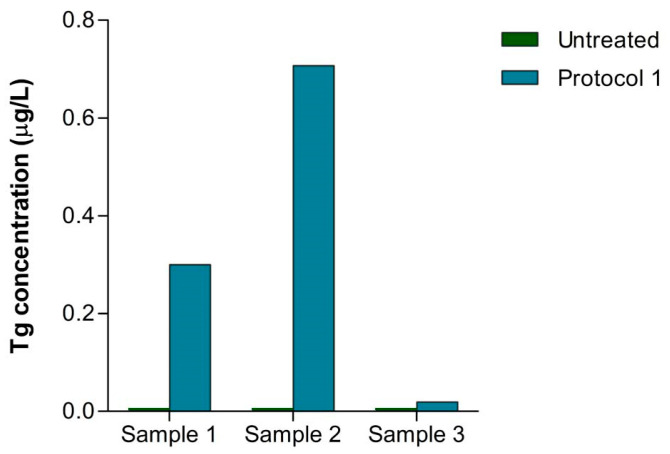
The effect of sample pre-processing on the Tg levels in patients with structural DTC recurrence (Sample 1) and follow up for DTC (Sample 2 and Sample 3).

Finally, we tested all preanalytical protocols on samples from three patients in follow up for DTC. Patient 1 had a confirmed structural recurrence, while patients 2 and 3 were considered to be in remission. Before the treatment, all samples were negative for Tg, and patients 2 and 3 were positive for TgAbs (1:700 and 1:2000, respectively). Although all tested protocols raised the Tg levels, we are presenting only the result after Protocol 1 pretreatment, as only Protocol 1 reached statistical significance in raising the Tg levels in TgAb-positive patients. After the preanalytical treatment of sera by Protocol 1, all samples exhibited a rise in Tg levels (Figure 4), while the rise was especially high in the serum sample that had a 1:700 TgAb titer. These results raise the possibility that patients 2 and 3 could have a biochemical recurrence of DTC.

## 3. Discussion

Detecting Tg in patients with recurrent disease and existing antibodies remains a challenging issue. While mass spectrometry can address this problem, it is not commonly used due to its high cost, limited effectiveness, and low throughput [26]. An alternative proposal involved using TgAb detection as a surrogate marker. However, this approach has not been widely adopted, as TgAb assays vary in sensitivity and absolute antibody levels [16,17], so even low TgAb concentrations considered “within the reference range” by the manufacturer can significantly interfere with Tg measurements. Furthermore, the variability in the time course of postoperative TgAb decline across individuals renders TgAb measurement an unreliable assessment [12,27,28,29]. Due to the variability in Tg and TgAbs caused by post-translational modification, no single assay can reliably predict interference [30]. Additionally, despite standardization against IRP 65/93, TgAb assays show considerable variability [18,31].

A promising approach involving preanalytical sample processing, until now, was explored solely by Kitamura et al. [20]. This group applied buffers and coated magnetic particles to capture Tg, which was afterwards chemiluminescently measured. To preserve the simplicity and cost effectiveness of traditional immunoassay tests, we employed a similar approach using only buffers (but with divergent composition) as a preanalytical protocol to treat samples prior to measuring Tg. Going a step further from using only chemical reagents, we applied additional mechanical pre-processing to separate Tg from TgAbs and, consequently, more accurately measure the Tg level.

Prior to starting the buffer selection phase, we evaluated the performance of the in-house IRMA test through interlaboratory comparison. The results demonstrated no significant deviation between the two methods and a high concordance rate with the reference laboratory, indicating strong agreement between the two techniques and confirming the robustness and reliability of our technique for subsequent experimental work.

The first phase of this study included the selection of the most suitable buffer, as its components can play a crucial role in stabilizing antigen–antibody interactions. While some may destabilize these interactions, others are designed to prevent degradation, maintain reactivity, and reduce non-specific interactions, thereby enhancing assay specificity [22,23,24,25,32]. It has been demonstrated that in a buffer with a pH of 8.8, i.e., in an alkaline environment, the interaction between Tg and TgAbs is weakened, allowing for the possibility of sugars and BSA to interact with the destabilized complex. BSA forms aggregates around both Tg and TgAbs, facilitating protein–protein interactions that help stabilize both protein fractions [33,34]. Additionally, the presence of sugars influences the surface tension of the solution, resulting in a shift in free energy. This change leads to the hydration of the proteins, thereby maintaining a stable microenvironment around them. This mechanism contributes to the stabilization of the proteins and enhances the overall system’s stability [23,35]. We tested five different buffers in two concentrations, among which only two did not produce false positive results, and the buffer that yielded the best results in raising Tg levels (10% 1B—Protocol 1) was selected for further evaluation. In addition to testing the 1B buffer as a preanalytical Protocol 1, micro-spin filters were also evaluated (as Protocol 2), both individually and in combination with the buffer (Protocol 3). It is important to note that the number of tested samples differed across Protocols 1, 2, and 3, as in some cases, there was an insufficient volume of samples available to conduct all analyses (as can be seen in Figure 3B). Statistical analysis revealed significant differences in measured Tg levels between untreated serums and serums treated by Protocol 1. This suggests that the preanalytical process might influence the release of Tg from the antigen–antibody complex, thereby enabling more accurate measurement of Tg values. Protocol 2, which included the use of micro-spin filter, did not significantly raise Tg levels. During the development of Protocol 2, several challenges were encountered. One major issue was the difficulty in standardizing the procedure to ensure uniform passage of all samples through the filter, which may have been affected by variations in sample viscosity, lipid content, and other factors. Additionally, it appears that large molecules, such as Tg, were likely retained on the filter. If the sample was not sufficiently detached from the filter by shaking after centrifugation, a lower Tg concentration was observed in those samples. However, a significant influence on Tg levels was observed during preanalytical processing with Protocol 3, which included the combination of buffer systems and the micro-spin filter, and this influence was stronger than when only buffer systems were employed (Protocol 1). However, due to challenges encountered in the technical implementation of Protocol 2, it appears that the enhanced effects of Protocol 3 vs. Protocol 1 can be attributed to concentrating the samples via a micro-spin filter rather than facilitating the release of the antigen–antibody complex. Moreover, the results obtained using both Protocols 1 and 3 showed a significant positive correlation to those obtained without treatment, suggesting that these protocols are consistent and compatible with the original test.

To further validate the hypothesis, the second phase of our study included an evaluation of the protocols in samples positive for TgAbs. The total sample was subdivided into three groups according to the level of TgAbs: those without TgAbs, those with low antibody titers, and those with high antibody titers. This approach allowed us to explore the impact of preanalytical processing across these distinct groups. Interestingly, when looking at Tg levels before treatment, Tg concentrations were generally lower in the group of patients without antibodies compared to those with low antibody titers, while the concentration decreased in the group with high antibody titers. This trend may be attributed to the possibility that patients with low antibody titers have higher Tg secretion, potentially due to the goiter/nodule’s occurrence [36,37]. The low antibody titer may not significantly interfere with Tg measurement, allowing for clearer detection of Tg levels in these patients. Conversely, in patients with high antibody titers, the detection of Tg might be more strongly hindered by the masking effect of the antibodies. In analyzing the results of preanalytical processing, the change in Tg measurement was presented as ratios between treated and untreated samples for easier monitoring. For all three tested protocols, we observed that the ratio exceeding 1, indicating an increase in measured Tg, generally rose, going from the group without TgAbs to those with a low titer, and it was the highest in cases with a high antibody titer. Statistical significance was observed in the change in Tg concentration using Protocol 1 across the three sample categories based on TgAb titer levels, and this is mainly due to the Tg concentration changes observed between the group of patients without antibodies and the group with a high antibody titer. This confirms our assumptions from the first phase of research, i.e., that treating the samples with buffer systems destabilizes the bond between Tg and TgAbs, releasing Tg from this complex and enabling its detection. While outliers were not excluded from the statistical analyses and graphical representations, it is important to note that in the case of Protocol 1, their presence may be attributable to the limited availability of serum, which restricted our ability to perform duplicate tests. In the case of Protocol 3, the difficulties encountered are likely due to technical issues and an incomplete protocol development, which led to Tg, as a large molecule, remaining adhered to the filter during the procedure.

In the final phase of the research, we analyzed samples from patients with recurrence (specifically, one confirmed case and two under follow up), all of which initially showed unmeasurable Tg levels. Following treatment, an increase in Tg concentration was observed in all three cases for Protocol 1, with the most pronounced rise noted in the patient with a high antibody titer. This observation further supports the effectiveness of this protocol as a more convenient and reliable method for measuring extremely low concentrations of Tg in the presence of antibodies compared to Protocols 2 and 3.

The primary limitation of our study is the relatively low number of tested samples. Additionally, we did not consider that spin filters may not be suitable for serum samples, making them challenging to standardize for this purpose. However, it is important to note that this was a pilot study aimed at assessing the feasibility of this approach. Consequently, some variables were not included in this initial phase. In considering the impact of temperature variation on measurements, we choose to perform the measurements at room temperature as the optimal temperature for influencing antigen–antibody interactions. For instance, hydrogen bonds, which are exothermic, generally stabilize at lower temperatures and are especially important when the antigen is a carbohydrate. Conversely, hydrophobic interactions tend to strengthen with increasing temperature. Consequently, while antibodies might be adapted to higher temperatures, antigen–antibody interactions are typically more stable at lower temperatures [32]. Taking this into consideration, we believe that temperature variations had a negligible effect on the test results.

## 4. Materials and Methods

### 4.1. Serum Samples and Study Groups

A total of 47 serum samples were used for this study. Among these, 44 were collected at INEP from patients who came for Tg and TgAb measurements, including those undergoing routine checks and those with various endocrinological conditions. Additionally, 3 samples were obtained from post-surgical PTC patients monitored for recurrence at the Clinic for Endocrinology, Diabetes, and Metabolic Diseases, Clinical Center of Serbia, Belgrade. Of these, one patient had structurally persistent disease, while the other two were in follow up for DTC and were considered to be in remission but had not yet returned for clinical evaluation at the time of writing the paper. All subjects had their Tg levels measured, while, due to the limited availability of the serum, TgAbs were measured in 45 patients. Further, due to the methodological difficulties during the processing, there were some additional exclusions of the patients (explained in detail in the Results section).

All patients included in this study were categorized into three groups based on antibody titer levels. The first group included patients with a negative antibody result (titer < 1:100)—13 cases; the second group comprised patients with moderate antibody levels (titer between 1:100 and 1:300)—12 cases; and the third group included patients with high antibody titers (titer > 1:300)—20 cases.

This study was conducted according to the guidelines of the Declaration of Helsinki, and all patients provided signed informed consent. Approval for the collection and use of human serum samples for this project was obtained from the local Ethics Committee of the INEP Institute (Approval No. 02-71/2) and the Ethics Committee of the University Clinical Centre of Serbia (Approval No. 140/15). All patients provided signed informed consent.

### 4.2. Thyroglobulin IRMA Assay

Determining Tg concentration was performed using a Tg IRMA assay (INEP, Belgrade, Serbia) standardized against human thyroglobulin reference material CRM 457 (Institute for the Reference Materials and Measurements IRMM, Geel, Belgium). This assay presents a solid-phase double (sandwich) immunoradiometric technique using two monoclonal antibodies specific for different epitopes of the Tg molecule. In short, 100 µL of sample volume and radiolabeled anti-Tg monoclonal antibody were added to the tubes coated with the second anti-Tg and incubated overnight. Upon washing, the radioactivity of the bound complex was counted on a gamma counter (Wallac Wizard 1470, Perkin Elmer, Waltham, MA, USA). The concentration of Tg in the sample is directly proportional to the measured amount of radioactivity and is expressed in µg/L. The detection limit of the assay is 0.05 µg/L; the measuring range is 0.1–200 µg/L, while the reference range for the healthy population is 0.5–40 µg/L.

### 4.3. Thyroglobulin Antibody RIA Assay

The concentration of thyroglobulin antibodies was measured using a TgAb radioimmunoassay (TgAb RIA, INEP, Belgrade, Serbia) standardized against the International Reference Preparation (IRP) MRC 65/93 (National Institute for Biological Standards and Control NIBSC, UK), which employs a 125I-labeled Tg molecule as a tracer. Briefly, radiolabeled Tg and 100 µL of the sample were added to the monoclonal anti-Tg-labeled RIA tubes and incubated for 2 h at RT. Upon washing, the immunocomplex was precipitated by polyethyleneglycol (PEG, Merck, Darmstadt, Germany), and the radioactivity was measured on a gamma counter (Wallac Wizard 1470, Perkin Elmer, Waltham, MA, USA). The results of the TgAb RIA are expressed as antibody titers; the detection limit of the assay is antibody titer 1:100, while the measuring range is antibody titers of 1:100–1:50,000. The reference range for the healthy population is antibody titer < 1:100.

### 4.4. Interlaboratory Comparison of Tg Immunoassay

In order to confirm the validity of INEPs Tg IRMA test, a bilateral comparison of Tg concentration was performed by applying Tg IRMA INEP’s registered test and a Beckman Coulter ACCESS immunoassay kit (Beckman Coulter Inc., Brea, CA, USA) in the Military Medical Academy biochemical laboratory.

### 4.5. Preanalytical Treatment Protocols

1. Protocol 1: preanalytical processing of serum using a buffer system
  Serum samples were separated from the blood cells by centrifugation, and 90 µL was used for analysis. After equilibrating the selected buffer at room temperature, 10 µL of the buffer system was mixed with 90 µL of serum and transferred into IRMA-coated test tubes. The samples were thoroughly mixed by vertexing, upon which the Tg measurement was directly performed. The final concentration of Tg was recalculated according to the dilution factor. The composition of two buffers tested for use in Protocol 1 was as follows: 1A buffer: 100 mM TBS, 5% BSA, 5% saccharose, and pH = 6.9; 1B buffer: 50 mM TBS, 5% BSA, 5% trehalose, and pH = 8.8. Additionally, upon final selection of the conditions for Protocol 1, this pretreatment was also tested for influencing the bond between Tg and Tg antibodies that are used in the IRMA test per se, and the materials and methods used for this analysis are included in Supplementary Data under Supplementary Materials and Methods.
2. Protocol 2: preanalytical processing of serum using micro-spin filters
  For this protocol, PVDF Micro Spin Filters (MWCO 200 kDa, 800 µL, with 2 mL receiver tubes) were purchased from Analytical, 179 Rt 206, Flanders, NJ, USA. Before using micro-spin filters, according to the manufacturer’s instructions, the filters (membrane) were soaked in deionized water (around 100 µL). Both preanalytically unprocessed and processed serum samples (200 µL each) were passed through filters. The samples in tubes were centrifuged at 15,000× *g*. The centrifugation time, determined empirically, varied from 1.5 to 10 min, depending on the sample characteristics, and lasted until 100 µL remained (the amount needed for the analytical measurement).
3. Protocol 3: preanalytical processing of serum using a combination of the buffer system and micro-spin filters
  This protocol mirrors the protocol for preanalytical processing using micro-spin filters, with the addition that the sample to be filtered is mixed with the selected buffer. Buffer 1B in 10% was used for this analysis.

### 4.6. Statistical Data Analysis

The normality of the distribution was verified using Shapiro–Wilk’s test for distribution type, as well as visual methods, such as the creation of relevant Q-Q plots and histograms. All the variables examined in this study had non-Gaussian distributions. As a result, a non-parametric set of tests was applied. The Wilcoxon signed-rank test and Spearman’s correlation test were used to compare Tg levels among laboratories. The Wilcoxon signed-rank test was used to assess the significance of each protocol’s effect on Tg change. The correlation between Tg levels measured before and after pre-processing was determined using Spearman’s test of correlation. To determine if the level of TgAbs in the patient’s serum has a significant effect on the effectiveness of the pretreatment, the Kruskal–Wallis test with a post hoc pairwise comparison was used. The results were statistically significant at *p* < 0.05. Statistical analysis was carried out using SPSS software (SPSS 16.0, Chicago, IL, USA) and GraphPad Prism 5 (GraphPad Software, Inc., La Jolla, CA, USA).

## 5. Conclusions

In conclusion, our study suggests that the preanalytical treatment of serum samples using a buffer in the presence of TgAbs may offer a promising approach for the follow up of DTC patients, particularly the recurrent ones. This method not only maintains the simplicity and cost effectiveness of immunoassay techniques but also avoids the complexities and expenses associated with low-throughput methods. Furthermore, we found that the use of spin filters is not suitable for any pre-treatment protocols involving serum samples. Future research should explore the optimization of these approaches to enhance their applicability in clinical settings.

## Figures and Tables

**Table 1:** Correlation analysis of the tested preanalytical protocols.

Spearman’s Correlation	Treatment	Protocol 1	Protocol 2	Protocol 3
r	**Untreated**	0.981 **	0.985 **	0.971 **
*p*		0.000	0.000	0.000
r	**Protocol 1**		0.984 **	0.978 **
*p*			0.000	0.000
r	**Protocol 2**			0.990 **
*p*				0.000

r—correlation coefficient, *p*—*p*-value, ** *p* < 0.001

## Data Availability

The raw data supporting the conclusions of this article will be made available by the authors upon request.

## Supplementary Materials

The following supporting information can be downloaded at: https://www.mdpi.com/article/10.3390/ijms252413252/s1.
