# Supplementary material for: Advancing Immunoassay Precision: A Novel Preanalytical Method for Enhancing Thyroglobulin Measurement in the Presence of Tg Antibodies
Source: Int J Mol Sci. 2024 Dec 10;25(24):13252. doi: 10.3390/ijms252413252 (PMC11675399; doi:10.3390/ijms252413252)
Supplement: Supplementary file 1 [file ijms-25-13252-s001.zip › ijms-3278732-supplementary.pdf]

Supplementary Data

Supplementary Material and Methods

Influence of Protocol 1 buffer system on thyroglobulin IRMA Assay

We prepared 4 serial dilutions of recombinant human thyroglobulin (rhTg, T6830, Sigma-Aldrich, Saint Louise, MO, USA) in the standard dilution buffer and measured the levels of rhTg with Tg IRMA INEP test. Next, we added the buffer used in Protocol 1 (1B, with the same percentage of 10%) to the rhTg serial dilutions and performed the second measurement of rhTg, which was then compared to the levels of rhTg in the samples free of Protocol 1 buffer.

Supplementary Figures

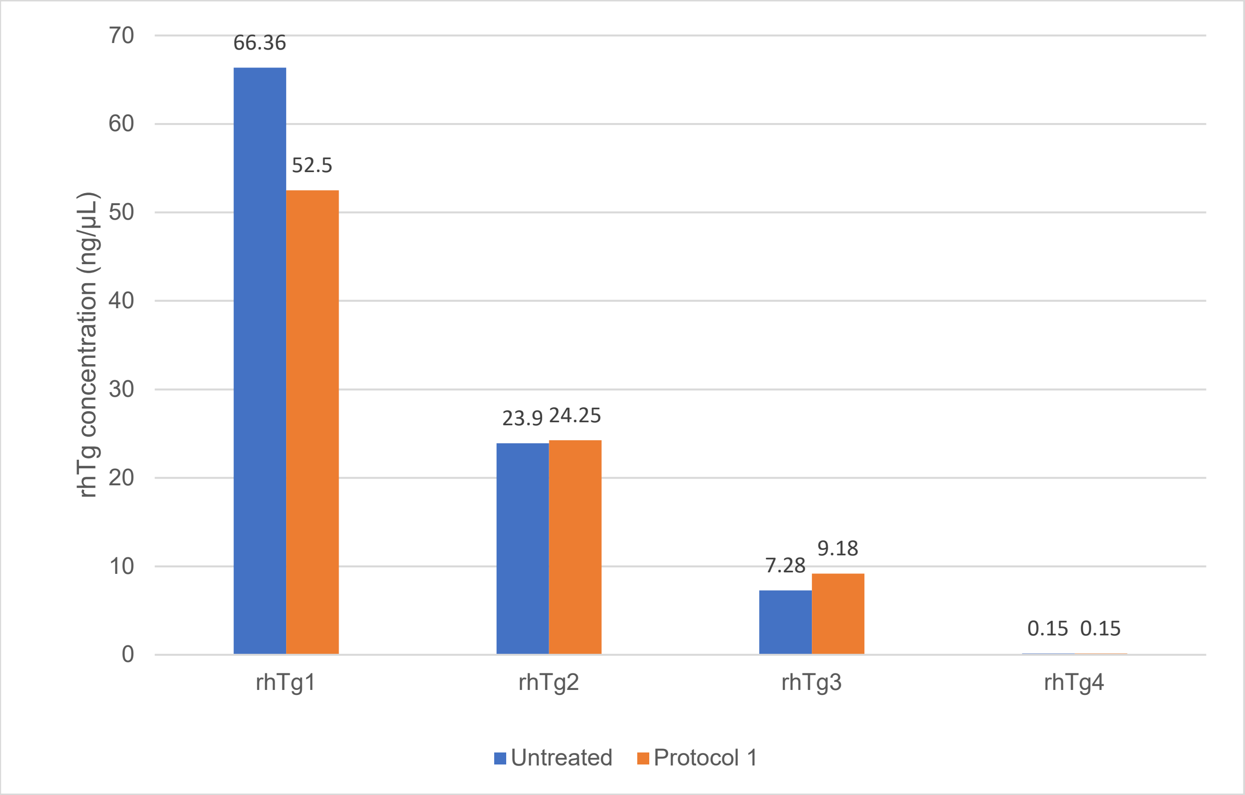

Figure S1. The concentration of recombinant human Tg (rhTg) measured in the samples prepared by diluting the rhTg in either standard buffer for Tg (Untreated) or standard buffer for Tg with the subsequent addition of disassociation buffer (Protocol 1).
